# Supplementary figures and images for: Evidence that a common arbuscular mycorrhizal network alleviates phosphate shortage in interconnected walnut sapling and maize plants
Source: Front Plant Sci. 2023 Aug 10;14:1206047. doi: 10.3389/fpls.2023.1206047 (PMC10448772; doi:10.3389/fpls.2023.1206047)

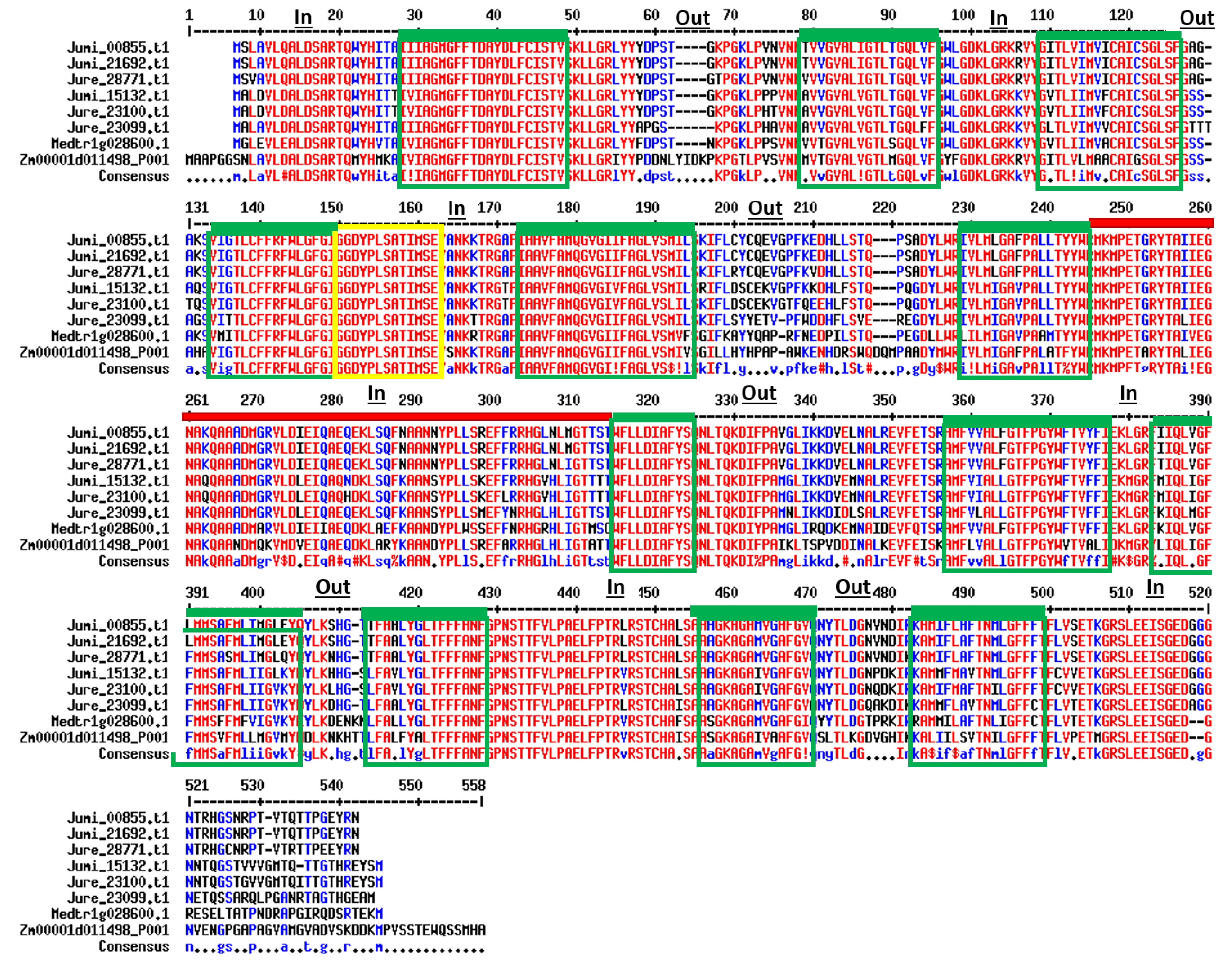

Supplement: Supplementary Figure 1 — Sequence alignment of the mycorrhiza-inducible phosphate transporter M. truncatula PT4 (Medtr1g028600.1) and its corresponding orthologs in J. regia (JrPHT1;1/Jr13_30200_p1, JrPHT1;2/Jr13_30210_p1; JrPHT1;3/Jr16_00830_p1), J. microcarpa (JmPHT1;1/jumi_00855.t1, JmPHT1;2/jumi_15132.t1; JmPHT1;3/jumi_21692.t1), and Z. mays (Zm00001d011498_P001). Protein sequences were aligned using MultAlin (http://multalin.toulouse.inra.fr/multalin/). Green bars indicate predicted transmembrane segments according to DeepTMHMM (https://dtu.biolib.com/DeepTMHMM). Yellow and red bars underline the sequence signature GGDYPLSATIxSE and the intracellular central loop conserved between PHT1 proteins, respectively. Cytoplasmic (In) and exoplasmic (Out) sequence orientations were predicted according to DeepTMHMM (https://dtu.biolib.com/DeepTMHMM). [file Image_1.tif]

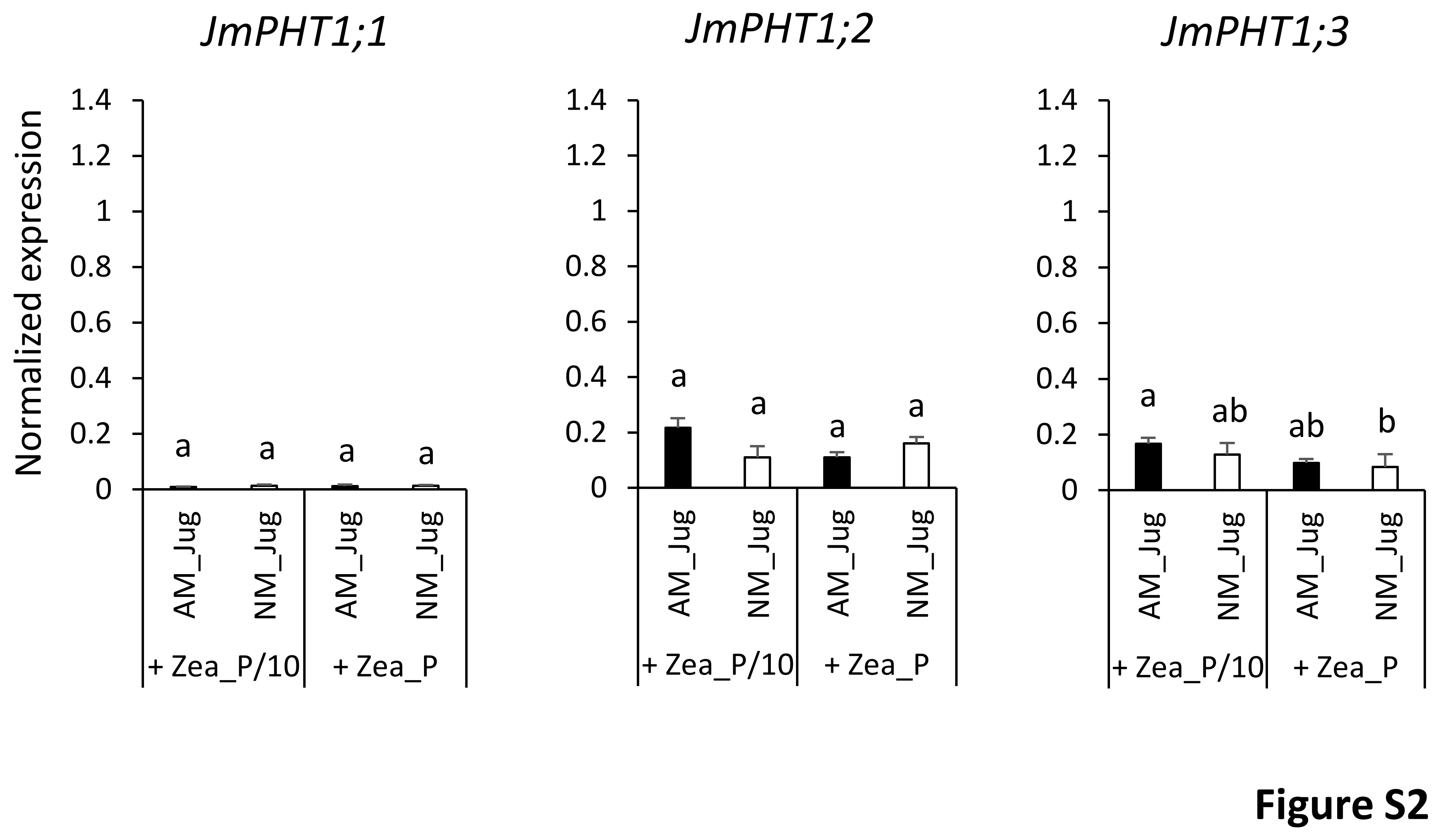

Supplement: Supplementary Figure 2 — Expression patterns of the genes JmPHT1;1; JmPHT1;2, JmPHT1;3 coding proteins orthologous to the phosphate transporter MtPT4 in donor walnut RX1 roots 2 months after their inoculation with R. irregularis (AM_Jug) or not (NM_Jug) under contrasting Pi fertilization regimes of maize (Zea_P/10, Zea_P). Gene expression was quantified by RT-qPCR and expressed as normalized expression to the walnut reference genes described in Table S1 . Values correspond to the mean ( ± SE) of five replicates per treatment. Different lowercase letters indicate significant difference (p < 0.05) according to the Kruskal–Wallis H-test with post-hoc Tukey HSD. [file Image_2.tif]
