# Supplementary material for: Evidence that a common arbuscular mycorrhizal network alleviates phosphate shortage in interconnected walnut sapling and maize plants
Source: Front Plant Sci. 2023 Aug 10;14:1206047. doi: 10.3389/fpls.2023.1206047 (PMC10448772; doi:10.3389/fpls.2023.1206047)
Supplement: Supplementary file 3 [file Table_1.docx]

**Table S1.** Primers used for qRT-PCR in *J. regia*, *J. microcarpa*, *Z. mais*, and *R. irregularis*

| **Oligoname** | **Sequence** | **Transcript ID/Reference** |
| --- | --- | --- |
| ***J. regia*** |  |  |
| *JregGAPDHrefF* | ATCATGGGTAAAGACCCCGC | XM_018984056.1/ Zhou et al. (2018) / doi: 10.1371/ journal.pone.0209424 |
| *JregGAPDHrefR* | TCAGTGACCGCATCCTTAGC |  |
| *JregACT2refF* | ATGGTCCCAAACATGACCCA | [XM_018959949.1/Zhou et al. (2018](https://www.ncbi.nlm.nih.gov/sites/entrez?cmd=Search&db=nucleotide&term=XM_018959949.1&dopt=GenBank))/ doi: 10.1371/ journal.pone.0209424 |
| *JregACT2refR* | TGTTGGAGGAGCTTGTGCAG |  |
| *JregPT4-28771F* | TCGAGAAGCTGGGTCGTTTC | Jr16_00830_p1/This study |
| *JregPT4-28771R* | TTCAGGAGTGGTCCGAGTCA |  |
| *JregPT4-1F* | GTTGCCCTTGTTGGTACCCT | Jr13_30200_p1/This study |
| *JregPT4-23100R* | AGCATATGGCGCAAAACACC |  |
| *JregPT4-23099F* | CCTGGCAGTAAGCCTGGAAA | Jr13_30210_p1 /This study |
| *JregPT4-23099R* | AGTTTATCACCCAGCCAGCC |  |
| ***J. microcarpa*** |  |  |
| *JmicGAPDHrefF* | CCCTCACAGCGAAACGACC | Jumi_01067.t1/This study |
| *JmicGAPDHrefR* | TTTTTGAAGAATGGTGATGCAGGC | |
| *JmicACT2refF* | ACGGGTGGGGAAGATTTGAC | Jumi_26547.t1/This study |
| *JmicACT2refR* | GGAGGGAGCACCATGTATCC |  |
| *JmicPT4-2192F* | ATGTCCCCGAACTCCTCTATCTT | Jumi_21692.t1/This study |
| *JmicPT4-2192R* | CGATTGTGTTGTCGCAGGG |  |
| *JmicPT4-15132F* | GCCCAAGACTCCTTAACCCC | Jumi_15132.t1/This study |
| *JmicPT4-15132R* | CATCGACGAGGCTGATACGA |  |
| *JmicPT4-855F* | AGAACCAGAGAACAGTGGCG | Jumi_00855.t1/This study |
| *JmicPT4-855R* | CATGGACCCTCACCTTCGAC |  |
| ***Z. mays*** |  |  |
| *Zm B-tubulinrefF* | CTACCTCACGGCATCTGCTATGT | Liu et al. (2014) / doi: 10.1371/journal.pone.0095445 |
| *Zm B-tubulinrefR* | GTCACACACACTCGACTTCACG |  |
| *Zm-EF1*αrefF | TGGGCCTACTGGTCTTACTACTGA | Liu et al. (2014)./ doi: 10.1371/journal.pone.0095445 |
| *Zm-EF1αrefF* | ACATACCCACGCTTCAGATCCT |  |
| *ZmPT1;6 F* | TTCTGCATCTCCACCGTGTC | Zm00001d011498_P001= ZmPht1;6 / Glassop et al. (2005) / doi: 10.1007/s00425-005-0015-0 |
| *ZmPT1;6 R* | CGCCTGTCACCATGTTGTTG |  |
| ***R. irregularis*** |  |  |
| *Riα-tubulin a1 F* | TGTCCAACCGGTTTTAAAGT | TC105406/Gomez et al. (2009) / doi: 10.1186/1471-2229-9-10 |
| *Riα-tubulin a1 R* | AAAGCACGTTTGGCGTACAT |  |
| *RiTEFrefF* | TGACAGGCGATCTGGTAAGG | Calabrese et al. (2019) / doi: 10.3389/fpls.2019.01617 |
| *RiTEFrefR* | TCAGCGAAGGTCTCAACCAC |  |
| *RiPT1F* | TGCTCGGTTGTGGTGCTATT | TC345640/Calabrese et al. (2019) / doi: 10.3389/fpls.2019.01617 |
| *RiPT1R* | CGACGTCCGAAGTTGCTTTG |  |
