# Supplementary material for: Evidence that a common arbuscular mycorrhizal network alleviates phosphate shortage in interconnected walnut sapling and maize plants
Source: Front Plant Sci. 2023 Aug 10;14:1206047. doi: 10.3389/fpls.2023.1206047 (PMC10448772; doi:10.3389/fpls.2023.1206047)
Supplement: Supplementary file 4 [file Table_2.docx]

**Table S2.** Effect of AM colonization and Pi availability on plant growth and nutritional parameters. Values correspond to the mean (±SE) of six replicates per treatment. Different lower case letters indicate significant difference (Kruskal-Wallis H-test with post-hoc Tukey HSD; *p* < 0.05).

|  | | Zea_P/10 | | Zea_P | |  |
| --- | --- | --- | --- | --- | --- | --- |
| ***Walnut*** | | AM | NM | AM | NM | pvalue |
| Root length (cm) | | 33.13±5.1 | 46.42±4.1 | 38.10±4.3 | 38.73±5.2 | 0.47 |
| **Collar** (mm) | | **3.78±0.3 ^ab^** | **2.93±0.2 ^c^** | **3.99±0.2 ^a^** | **3.04±0.2 ^bc^** | **<0.01** |
| **Stem height** (cm) | | **17.02±1.7 ^a^** | **10.10±1.9 ^b^** | **12.84±0.8 ^ab^** | **10.10±1.2 ^b^** | **0.02** |
| Branch number | | 5.17±0.7 | 4.50±0.5 | 4.29±0.4 | 5.14±0.5 | 0.56 |
| **Shoot DW** (g) | | **1.26±0.2 ^a^** | **0.51±0.1 ^b^** | **1.06±0.1 ^a^** | **0.57±0.1 ^b^** | **<0.01** |
| **Root DW** (g) | | **2.50±0.3 ^a^** | **0.81±0.2 ^b^** | **1.85±0.2 ^a^** | **0.94±0.3 ^b^** | **<0.01** |
| **Shoot/root** | | **0.49±0.03 ^b^** | **0.86±0.2 ^a^** | **0.58±0.03 ^ab^** | **0.68±0.1 ^a^** | **0.05** |
| Fv_fm | | 0.72±0.02 | 0.71±0.02 | 0.70±0.01 | 0.71±0.01 | 0.90 |
| **Y_II_** | | **0.45±0.01 ^a^** | **0.34±0.02 ^b^** | **0.44±0.01 ^a^** | **0.42±0.03 ^a^** | **<0.01** |
| Root N (%DW) | | 1.5±0.1 | 1.6±0.1 | 1.7±0.1 | 1.7±0.1 | 0.33 |
| **Root C** (%DW) | | **39.6±0.3 ^b^** | **42.8±0.3 ^a^** | **39.9±1.4 ^b^** | **40.3±0.1 ^b^** | **0.02** |
| Root Pi (nmole/mg) | | 40.18±4.2 | 45.97±6.3 | 50.56±2.6 ^a^ | 41.82±3.1 ^a^ | 0.13 |
| Leaf N (%DW) | | 2.8±0.1 | 2.7±0.1 ^a^ | 2.6±0.04 ^a^ | 2.8±0.1 ^a^ | 0.18 |
| Leaf C (%DW) | | **45.4±0.3 ^a^** | **44.3±0.4 ^ab^** | **44.5±0.1 ^ab^** | **43.8±0.4 ^b^** | **0.04** |
| **Leaf Pi** (nmole/ mg DW) | | **62.0±1.6 ^a^** | **49.1±4.7 ^b^** | **53.3±4.5 ^ab^** | **65.9±5.4 ^a^** | **0.03** |
| Leaf C/N | | 16.2±0.5 | 16.6±0.6 | 17.3±0.3 | 15.6±0.9 | 0.3 |
|  | Zea_P/10 | | | Zea_P | |  |
| ***Maize*** | AM | | NM | AM | NM | pvalue |
| Root length (cm) | 59.0±4.2 | | 45.3±2.5 | 53.7±2.4 | 60.7±6.7 | 0.05 |
| **Collar** (mm) | **16.8±0.4 ^a^** | | **12.3±0.9 ^b^** | **17.4±0.4 ^a^** | **16.8±0.8 ^a^** | **<0.01** |
| **Stem height** (cm) | **135.5±8.8^a^** | | **82.6±10.8 ^b^** | **132.1±11.1 ^a^** | **129.9±12.3 ^a^** | **0.02** |
| Leaf number | **8.2±0.3 ^a^** | | **6.6±0.3 ^b^** | **8.6±0.2^a^** | **8.4±0.2^a^** | **<0.01** |
| **Shoot DW** (g) | **3.6±0.7 ^a^** | | **2.4±0.4 ^b^** | **6.8±1.0 ^a^** | **5.6±0.7 ^a^** | **<0.01** |
| **Root DW** (g) | **1.2±0.1 ^a^** | | **0.4±0.1 ^b^** | **0.9±0.1^a^** | **0.9±0.1 ^a^** | **<0.01** |
| Shoot/root | **5.6±0.5 ^b^** | | **5.5±0.5 ^b^** | **8.1±0.6 ^a^** | **6.7±0.7 ^ab^** | **0.04** |
| Fv_fm | **0.77±0.01 ^ab^** | | **0.76±0.01 ^a^** | **0.77±0.01 ^ab^** | **0.78±0.01^b^** | **<0.01** |
| Y_II_ | 0.46±0.01 | | 0.45±0.01 | 0.43±0.01 | 0.46±0.01 | 0.3 |
| Root N (%DW) | 1.7±0.2 | | 1.7±0.1 | 1.5±0.2 | 1.7±0.1 | 1.0 |
| Root C (%DW) | 31.4±2.5 | | 34.4±1.6 | 32.9±2.7 | 33.7±1.8 | 0.8 |
| Root Pi (nmole Pi/mg DW) | | 66.3±7.7 | 42.9±1.9 | 52.4±3.3 | 47.6±8.1 | 0.1 |
| **Leaf N** (%DW) | **2.9±0.1 ^a^** | | **2.6±0.1 ^b^** | **3.0±0.1 ^a^** | **2.9±0.2 ^a^** | **0.02** |
| Leaf C (%DW) | 44.1±0.3 | | 44.1±0.1 | 44.3±0.2 | 44.7±0.2 | 0.1 |
| **Leaf Pi** (nmole Pi/mg DW) | | **102.19±5.6 ^a^** | **79.3±2.6 ^b^** | **123.8±7.2 ^a^** | **115.7±11.4 ^a^** | **<0.01** |
| **Leaf C/N** | **15.1±0.5 ^b^** | | **17.1±0.4 ^a^** | **14.8±0.4^b^** | **15.6±0.9 ^ab^** | **0.02** |
