# Supplementary material for: Evidence that a common arbuscular mycorrhizal network alleviates phosphate shortage in interconnected walnut sapling and maize plants
Source: Front Plant Sci. 2023 Aug 10;14:1206047. doi: 10.3389/fpls.2023.1206047 (PMC10448772; doi:10.3389/fpls.2023.1206047)
Supplement: Supplementary file 5 [file Table_3.docx]

**Table S3**. Proteins of *J. microcarpa,* *J. regia,* and *Z. mays* contained in the orthogroup of the mycorrhiza-inducible PHT1 transporter

MtPT4 of *M. truncatula* (**A**)*,* and additional plant PHT1 transporters used to build the PHT1 phylogenic tree (**B**).

| **Protein name** | **Organism** | **Accession** | **Reference** | **Source** |
| --- | --- | --- | --- | --- |
| **A** |  |  |  |  |
| JmPHT1;1 | *Juglans microcarpa* | jumi_00855.t1 | This study | https://treegenesdb.org/FTP/Genomes/Jumi/v1.0/annotation/jumi.1_0.peptides.fa |
| JmPHT1;2 | *Juglans microcarpa* | jumi_15132.t1 | This study | https://treegenesdb.org/FTP/Genomes/Jumi/v1.0/annotation/jumi.1_0.peptides.fa |
| JmPHT1;3 | *Juglans microcarpa* | jumi_21692.t1 | This study | https://treegenesdb.org/FTP/Genomes/Jumi/v1.0/annotation/ jumi.1_0.peptides.fa |
| JrPHT1;1 | *Juglans regia* | Jr13_30200_p1, A0A2I4GU17 | This study | https://treegenesdb.org/FTP/Genomes/Jumi/v1.0/annotation/jumi.1_0.peptides.fa |
| JrPHT1;2 | *Juglans regia* | Jr13_30210_p1, A0A2I4GU05 | This study | https://treegenesdb.org/FTP/Genomes/Jumi/v1.0/annotation/jumi.1_0.peptides.fa |
| JrPHT1;3 | *Juglans regia* | Jr16_00830_p1, A0A6P9EI50 | This study | https://treegenesdb.org/FTP/Genomes/Jumi/v1.0/annotation/ jumi.1_0.peptides.fa |
| MtPT4 | *Medicago truncatula* | Q8GSG4 | Harrison et al., 2002 | https://doi.org/10.1105/tpc.004861  https://genome.jgi.doe.gov/portal/pages/dynamicOrganismDownload.jsf?organism=Mtruncatula/Mtruncatula_198_protein.fa.gz |
| ZmPT6 | *Zea mays* | Zm00001d011498_P001/ NP_001105776.1 | Liu et al., 2016 | <https://ftp.ensemblgenomes.org/pub/release-43/plants/fasta/zea_mays/pep/>Zea_mays.B73_RefGen_v4.pep.all.fa.gz; https://www.ncbi.nlm.nih.gov/ |
| **B** |  |  |  |  |
| AtPT1 | *Arabidopsis thaliana* | NP_199149.1 | Tabata et al., 2000 | https://doi.org/10.1038/35048507 |
| AtPT2 | *Arabidopsis thaliana* | NP_001190462.1 | Tabata et al., 2000 | https://doi.org/10.1038/35048507 |
| AtPT3 | *Arabidopsis thaliana* | NP_199150.1 | Tabata et al., 2000 | https://doi.org/10.1038/35048507 |
| AtPT4 | *Arabidopsis thaliana* | NP_181428.1 | Lin et al., 1999 | https://doi.org/10.1038/45471 |
| AtPT5 | *Arabidopsis thaliana* | NP_180842.1 | Lin et al., 1999 | https://doi.org/10.1038/45471 |
| AtPT6 | *Arabidopsis thaliana* | NP_199148.1 | Tabata et al., 2000 | https://doi.org/10.1038/35048507 |
| AtPT7 | *Arabidopsis thaliana* | NP_001319749.1 | Salanoubat et al, 2000 | https://doi.org/10.1038/35048706 |
| AtPT8 | *Arabidopsis thaliana* | NP_001323235.1 | Theologis et al., 2000 | https://doi.org/10.1038/35048500 |
| AtPT9 | *Arabidopsis thaliana* | NP_177769.1 | Theologis et al., 2000 | https://doi.org/10.1038/35048500 |
| BdPT7 | *Brachypodium distachyon* | XP_003569484.1 | Hong et al. 2002 | https://doi.org/10.1007/s00425-012-1677-z |
| EcPT1 | *Eleusine coracana* | AJD86034 | Pudake et al., 2017 | https://doi.org/10.1007/s13205-017-0609-9 |
| EcPT2 | *Eleusine coracana* | AJD86035.1 | Pudake et al., 2017 | https://doi.org/10.1007/s13205-017-0609-9 |
| EcPT3 | *Eleusine coracana* | AJD86036.1 | Pudake et al., 2017 | https://doi.org/10.1007/s13205-017-0609-9 |
| EcPT4 | *Eleusine coracana* | AJD86037.1 | Pudake et al., 2017 | https://doi.org/10.1007/s13205-017-0609-9 |
| GmPT1 | *Glycine max* | NP_001239971.1 | Fan et al., 2013 | https://doi.org/10.1186/1471-2229-13-48 |
| GmPT2 | *Glycine max* | NP_001240239.1 | Fan et al., 2013 | https://doi.org/10.1186/1471-2229-13-48 |
| GmPT3 | *Glycine max* | NP_001241164.1 | Fan et al., 2013 | https://doi.org/10.1186/1471-2229-13-48 |
| GmPT4 | *Glycine max* | NP_001304639.2 | Fan et al., 2013 | https://doi.org/10.1186/1471-2229-13-48 |
| GmPT5 | *Glycine max* | NP_001304588.2 | Wang et al., 2019 | https://doi.org/10.1186/s12870-019-1959-8 |
| GmPT6 | *Glycine max* | NP_001240032.1 | Fan et al., 2013 | https://doi.org/10.1186/1471-2229-13-48 |
| GmPT7 | *Glycine max* | NP_001239765.1 | Chen et al., 2019 | https://doi.org/10.1111/nph.15541 |
| GmPT8 | *Glycine max* | NP_001345390.1 | Fan et al., 2013 | https://doi.org/10.1186/1471-2229-13-48 |
| GmPT9 | *Glycine max* | NP_001241574.1 | Fan et al., 2013 | https://doi.org/10.1186/1471-2229-13-48 |
| GmPT10 | *Glycine max* | NP_001241400.1 | Fan et al., 2013 | https://doi.org/10.1186/1471-2229-13-48 |
| GmPT11 | *Glycine max* | NP_001241127.1 | Fan et al., 2013 | https://doi.org/10.1186/1471-2229-13-48 |
| HvPT1 | *Hordeum vulgare* | AAN37900.1 | Rae et al., 2003 | https://doi.org/10.1023/B:PLAN.0000009259.75314.15 |
| HvPT2 | *Hordeum vulgare* | AAO72434.1 | Smith et al., 1999 | https://doi.org/10.1007/978-94-017-2685-6_19 |
| HvPT4 | *Hordeum vulgare* | AAO72437.1 | Smith et al., 1999 | https://doi.org/10.1007/978-94-017-2685-6_19 |
| HvPT5 | *Hordeum vulgare* | AAO72435.1 | Smith et al., 1999 | https://doi.org/10.1007/978-94-017-2685-6_19 |
| HvPT6 | *Hordeum vulgare* | AAN37901.1 | Smith et al., 1999 | https://doi.org/10.1007/978-94-017-2685-6_19 |
| HvPT7 | *Hordeum vulgare* | AAO72436.1 | Smith et al., 1999 | https://doi.org/10.1007/978-94-017-2685-6_19 |
| HvPT8 | *Hordeum vulgare* | AAO72440.1 | Smith et al., 1999 | https://doi.org/10.1007/978-94-017-2685-6_19 |
| HvPT9 | *Hordeum vulgare* | AM904733.1 | Huag et al., 2011 | https://doi.org/10.1104/pp.111.178459 |
| LbPT1 | *Lycium barbarum* | AIU41746.1 | Hu et al., 2017 | https://doi.org/10.1093/treephys/tpw125 |
| LbPT2 | *Lycium barbarum* | AIU41747.1 | Hu et al., 2017 | https://doi.org/10.1093/treephys/tpw125 |
| LbPT3 | *Lycium barbarum* | AIU41748.1 | Hu et al., 2017 | https://doi.org/10.1093/treephys/tpw125 |
| LbPT4 | *Lycium barbarum* | AIU41749.1 | Hu et al., 2017 | https://doi.org/10.1093/treephys/tpw125 |
| LbPT5 | *Lycium barbarum* | AIU41750.1 | Hu et al., 2017 | https://doi.org/10.1093/treephys/tpw125 |
| LbPT7 | *Lycium barbarum* | AIU41751.1 | Hu et al., 2017 | https://doi.org/10.1093/treephys/tpw125 |
| LePT1 | *Lycopersicum esculentum* | CAA74607.1 | Daram et al., 1998 | https://doi.org/10.1007/s004250050394 |
| LePT2 | *Lycopersicum esculentum* | NP_001234043.1 | Liu et al., 1998 | https://doi.org/10.1104/pp.116.1.91 |
| LePT3 | *Lycopersicum esculentum* | NP_001318089.1 | Aoki et al., 2010 | https://doi.org/10.1186/1471-2164-11-210 |
| LePT4 | *Lycopersicum esculentum* | NP_001234674.2 | Nagy et al., 2005 | https://doi.org/10.1111/j.1365-313X.2005.02364.x |
| LePT5 | *Lycopersicum esculentum* | AAX85194.1 | Nagy et al., 2005 | https://doi.org/10.1111/j.1365-313X.2005.02364.x |
| LePT6 | *Lycopersicum esculentum* | AJF19139.1 | Chen et al., 2014 | https://doi.org/10.1186/1471-2229-14-61 |
| LePT7 | *Lycopersicum esculentum* | AJF19140.1 | Chen et al., 2014 | https://doi.org/10.1186/1471-2229-14-61 |
| LePT8 | *Lycopersicum esculentum* | AJF19141.1 | Chen et al., 2014 | https://doi.org/10.1186/1471-2229-14-61 |
| LjPT3 | *Lotus japonicus* | BAE93353.1 | Maeda et al., 2006 | https://doi.org/10.1093/pcp/pcj069 |
| LjPT4 | *Lotus japonicus* | BAG71408.1 | Takeda et al., 2009 | https://doi.org/10.1111/j.1365-313X.2009.03824.x |
| MtPT1 | *Medicago truncatula* | AAB81346.1 | Liu et al., 1998 | https://doi.org/10.1094/MPMI.1998.11.1.14 |
| MtPT3 | *Medicago truncatula* | ABM69110.1 | Liu et al., 1998 | https://doi.org/10.1094/MPMI.1998.11.1.14 |
| MtPT5 | *Medicago truncatula* | ABM69111.1 | Liu et al., 1998 | https://doi.org/10.1094/MPMI.1998.11.1.14 |
| MtPT6 | *Medicago truncatula* | XP_003601529.1 | Breuillin-Sessoms et al., 2015 | https://doi.org/10.1105/tpc.114.131144 |
| OsPT1 | *Oryza sativa* | Q8H6H4 | Paszkowski et al., 2002 | https://doi.org/0.1073/pnas.202474599 |
| OsPT2 | *Oryza sativa* | Q8GSD9 | Paszkowski et al., 2002 | https://doi.org/0.1073/pnas.202474599 |
| OsPT3 | *Oryza sativa* | Q7XDZ7 | Paszkowski et al., 2002 | https://doi.org/0.1073/pnas.202474599 |
| OsPT4 | *Oryza sativa* | Q8H6H2 | Paszkowski et al., 2002 | https://doi.org/0.1073/pnas.202474599 |
| OsPT5 | *Oryza sativa* | Q7X7V2 | Paszkowski et al., 2002 | https://doi.org/0.1073/pnas.202474599 |
| OsPT6 | *Oryza sativa* | Q8H6H0 | Paszkowski et al., 2002 | https://doi.org/0.1073/pnas.202474599 |
| OsPT7 | *Oryza sativa* | Q8H6G9 | Paszkowski et al., 2002 | https://doi.org/0.1073/pnas.202474599 |
| OsPT8 | *Oryza sativa* | Q8H6G8 | Paszkowski et al., 2002 | https://doi.org/0.1073/pnas.202474599 |
| OsPT9 | *Oryza sativa* | Q8H6G7 | Paszkowski et al., 2002 | https://doi.org/0.1073/pnas.202474599 |
| OsPT10 | *Oryza sativa* | Q69T94 | Paszkowski et al., 2002 | https://doi.org/0.1073/pnas.202474599 |
| OsPT11 | *Oryza sativa* | Q94DB8 | Paszkowski et al., 2002 | https://doi.org/0.1073/pnas.202474599 |
| PtPT10 | *Populus trichocarpa* | XP_006374329 | Loth-Pereda et al., 2011 | https://doi.org/10.1104/pp.111.180646 |
| SbPT1 | *Sorghum bicolor* | NP_001275200.1 | Leggewie et al., 1997 | https://doi.org/10.1105/tpc.9.3.381 |
| SbPT3 | *Sorghum bicolor* | CAC87043.1 | Rausch et al., 2001 | https://doi.org/10.1038/35106601 |
| SbPT11 | *Sorghum bicolor* | XP_002458253.1 | Walder et al., 2015 | https://doi.org/10.1111/nph.13292 |
| StPT2 | *Solanum tuberosum* | CAA67396.1 | Leggewie et al., 1997 | https://doi.org/10.1105/tpc.9.3.381 |
| StPT4 | *Solanum tuberosum* | AAW51149.1 | Karandashov and Bucher, 2005 | https://doi.org/10.1016/j.tplants.2004.12.003 |
| StPT5 | *Solanum tuberosum* | AAX85195.1 | Nagy et al., 2005 | https://doi.org/10.1111/j.1365-313X.2005.02364.x |
| TaPT1 | *Triticum aestivum* | CAC69856.1 | Davies et al., 2002 | https://doi.org/10.1046/j.1365-3040.2002.00913.x |
| TaPT8 | *Triticum aestivum* | AAP49822.1 | Chang et al., 2003 | unpublished |
| TmPT1 | *Triticum monococcum* | AAQ06280.1 | Ma et al., 2002 | unpublished |
| VvPT1 | *Vitis vinifera* | XP_002267369.1 | Valat et al., 2018 | https://doi.org/10.1007/s00572-017-0809-5 |
| VvPT2 | *Vitis vinifera* | XP_002267327.1 | Valat et al., 2018 | https://doi.org/10.1007/s00572-017-0809-5 |
| ZmPT1 | *Zea mays* | NP_001105269.2 | Wright et al., 2005 | https://doi.org/10.1111/j.1469-8137.2005.01472.x |
| ZmPT2 | *Zea mays* | NP_001105816.1 | Schnable et al., 2009 | https://doi.org/10.3390/ijms17060930 |
| ZmPT3 | *Zea mays* | AAY42387.1 | Nagy et al., 2005 | unpublished |
| ZmPT4 | *Zea mays* | AAY42388.1 | Nagy et al., 2005 | unpublished |
| ZmPT5 | *Zea mays* | ACG37120.1 | Alexendrov et al., 2009 | https://doi.org/10.1007/s11103-008-9415-4 |
